# Systemic Lupus Erythematosus Presenting With Cold-Antibody Autoimmune Hemolysis and Nephritis: A Case Report

**DOI:** 10.7759/cureus.67148

**Published:** 2024-08-18

**Authors:** Gabriel Calderon-Valverde, Mariana Quiros-Meza, Alberto Alfaro-Murillo

**Affiliations:** 1 Rheumatology, Universidad de Costa Rica - Hospital San Juan de Dios, San Jose, CRI; 2 Internal Medicine, Universidad de Costa Rica - Hospital San Juan de Dios, San Jose, CRI

**Keywords:** rituximab, lupus nephritis, immune hemolytic anemia, cold agglutinin syndrome, systemic lupus erythematosus

## Abstract

Systemic lupus erythematosus (SLE) is a multifaceted autoimmune disorder that presents with a wide array of clinical features, including autoimmune hemolysis and nephritis. Autoimmune hemolysis in SLE is typically linked to warm antibodies, but the occurrence of cold agglutinin syndrome (CAS), driven by cold-reactive antibodies, is exceptionally rare. Lupus nephritis (LN) is among the most severe complications of SLE, characterized by immune complex-mediated glomerulonephritis, which often leads to considerable morbidity and mortality. Nephritis in SLE is a major indicator of chronic kidney disease, with many patients experiencing progressive renal damage over time. Early diagnosis and individualized treatment approaches are crucial for effectively managing these intertwined conditions.

This case report presents a distinct clinical scenario involving a 53-year-old Hispanic female diagnosed with SLE, who concurrently presented with CAS and nephritis. The patient's initial symptoms included chest pain, severe macrocytic anemia, elevated creatinine levels, and evidence of active hemolysis. CAS was diagnosed through a positive direct antiglobulin test for C3d and elevated cold agglutinin titers. Further comprehensive assessments revealed dysgammaglobulinemia, hypocomplementemia, and positive anti-Ro antibodies, with a renal biopsy confirming LN (ISN/RPS Class IV and Class V).

The patient exhibited a favorable response to a treatment regimen comprising high-dose steroids and anti-CD20 therapy, resulting in the complete cessation of hemolysis and a >50% decrease in proteinuria after six months. This case underscores the rarity of CAS in the context of SLE, particularly when coupled with nephritis, and highlights the need for tailored treatment strategies. Anti-CD20 therapy, as used in primary CAS management, emerges as a promising option for this unique presentation, offering insights into the complex interplay of autoimmune conditions.

## Introduction

Systemic lupus erythematosus (SLE) is a chronic multisystemic autoimmune disease with complex innate and adaptive immunity interactions. It is characterized by important clinical heterogeneity, and its most common manifestations involve cutaneous, articular, renal, and hematological complications [1].

Autoimmune hemolytic anemia (AIHA) occurs in approximately 10% of patients with SLE, rarely manifesting as the presenting feature of the disease [2]. AIHA is associated strongly with positive direct antiglobulin tests (DAT) for warm-type Immunoglobulin G (IgG) antibodies in most cases. Cold agglutinin syndrome (CAS), caused by Immunoglobulin M (IgM) antibodies that agglutinate at low temperatures, is exceptionally rare in SLE with few cases being reported in the literature [3,4]. Although AIHA is a relatively rare manifestation, it can precede the diagnosis of SLE or be one of its presenting features. SLE-associated AIHA predominantly affects females and can manifest at any age, although it is more common in younger individuals [2]. 

Patients with SLE who develop AIHA typically present with symptoms of anemia, such as fatigue, pallor, and dyspnea, along with signs of hemolysis, including jaundice, dark urine, and splenomegaly. Laboratory findings often include normocytic anemia with elevated levels of lactate dehydrogenase (LDH), indirect hyperbilirubinemia, increased reticulocyte count, and reduced haptoglobin. Peripheral blood smears may show spherocytes [2]. 

The cornerstone of treatment for AIHA in SLE is high-dose glucocorticoids; for patients with severe, rapidly progressive hemolysis, intravenous methylprednisolone may be administered at doses ranging from 250 to 1,000 mg/day for one to three days [5]. Despite initial treatment, about one-third of patients do not achieve long-term remission, necessitating second-line therapies. Rituximab, a monoclonal anti-CD20 antibody, has gained prominence as a preferred second-line treatment due to its efficacy [6]. Rituximab has shown an overall response rate of approximately 79% in refractory or relapsed cases, although the response may take weeks to become evident, with relapse rates between 25% and 50% within one to two years. The differential diagnosis for SLE-associated AIHA includes other causes of hemolytic anemia, such as microangiopathic hemolytic anemias (e.g., thrombotic thrombocytopenic purpura, hemolytic uremic syndrome), drug-induced hemolytic anemia, and infections [2,7]. 

Lupus nephritis (LN) constitutes one of the most severe organ manifestations of SLE. About 25-50% of patients with SLE show signs of kidney disease at diagnosis, and up to 60% develop renal involvement during their disease course. LN is more prevalent in male SLE patients (27-75%) compared to females (16-52%), and it tends to be more common in juvenile-onset SLE (50-82%) than adult-onset SLE (34-53%). LN prevalence is higher among African American, Hispanic, and Asian populations compared to white individuals. Factors such as younger age, male sex, and African, Asian, or Hispanic ethnicity are associated with a higher likelihood of developing LN [8].

If LN is suspected, a kidney biopsy should be considered in patients with any degree of renal involvement, which is defined by the presence of glomerular hematuria and cellular casts, proteinuria >0.5g/24 hours, a proteinuria/creatininuria ratio >500mg/g, an unexplained decrease in glomerular filtration rate, and the presence of active urinary sediment (pathological casts, microscopic hematuria, leukocyturia) [1,8].

Currently, EULAR guidelines recommend mycophenolate mofetil (targeting 2-3 g/day) and cyclophosphamide in the Eurolupus regimen (500 mg every two weeks for six doses) as first-line induction therapy [1,9-11]. Between 5% and 20% of patients with LN develop chronic kidney disease within 10 years of their diagnosis, and the presence of kidney damage is the most important predictor of mortality in patients with SLE [12].

## Case presentation

A 53-year-old Hispanic female patient with a history of hypertension, autoimmune hypothyroidism, and dyslipidemia attended the emergency department due to worsening chest pain.

The patient had been followed up and treated in the primary care setting due to a five-year-old history of metabolic syndrome. One year ago, the patient began treatment with oral iron supplementation as moderate anemia had been diagnosed during routine check-ups. Six months before presentation, the patient noticed unintentional weight loss, night sweats, and fatigue; during the following months, symptoms worsened and eventually developed into moderate exertional dyspnea and lower-limb edema. The patient denied any skin rashes, mucosal compromise, or articular pain.

During emergency department evaluation, the patient complained about intense, oppressive retrosternal sudden-onset chest pain which radiated to her lower jaw. Her temperature was 36.8°C, heart rate 101 beats/min, and blood pressure 154/86 mmHg. She had bilateral lower limb and palpebral edema. ECG exhibited sinus tachycardia with otherwise normal findings. Complete blood count demonstrated profound macrocytic anemia: Hemoglobin 3.2 g/dL and mean corpuscular volume (MCV) 121 fL, and normal leukocyte and platelet count; creatinine levels were elevated Cr: 1.48 mg/dL without electrolyte imbalances; there were indirect signs of active hemolysis identified with elevated indirect bilirubin (IB) levels: 1.9 mg/dL, elevated lactate dehydrogenase (LDH) 550 IU/ml, elevated reticulocyte index (RI): 5.6 and indetectable haptoglobin levels: < 10 mg/dL. A direct antiglobulin test was positive for C3d with elevated cold agglutinin titers, and red blood cell (RBC) agglutination was observed at 4°C. The patient’s troponin T levels were also markedly elevated at 1163 pg/mL. Additional tests, including serial electrocardiograms, a trans-thoracic echocardiogram, and a pulmonary computed tomography angiogram, didn't reveal evidence of pulmonary embolism, left ventricular dysfunction, or coronary artery disease. 

Immediate RBC transfusion was initiated, and she was hospitalized for further analysis. Upon admission, the patient commenced treatment with oral prednisone at a dose of 1 mg/kg and received weekly rituximab at a dosage of 375 mg/m^2^. This regimen promptly normalized her hemoglobin levels, and there was no further evidence of hemolysis. Subsequent patient evaluation revealed normal levels of Vitamin B12, folate, and iron, but with elevated ferritin levels. Infectious causes, such as viral triggers of CAS, were ruled out through negative results for HIV, Hepatitis B, Hepatitis C, Mycoplasma pneumoniae, Epstein-Barr, and Cytomegalovirus serologies and viral loads. Similarly, hematological malignancy or dyscrasia studies were conducted, including a bone marrow biopsy and cellular flow cytometry, all of which failed to indicate any hematological malignancy or dyscrasia, and nephrotic range proteinuria was identified during 24-hour urine analysis (7.5 g/24h). Immunological evaluation demonstrated dysgammaglobulinemia, with elevated IgM (2.42 g/L) and normal IgA and IgG levels without monoclonal spikes in serum protein electrophoresis; hypocomplementemia (C3: 68 mg/dL and C4 4 mg/dL); Hep-2 indirect immunofluorescence was intensely positive at 1:3200 titers for anticellular antibodies with AC-4 and AC-5 patterns being reported, as well as positive Anti-Ro52 and Anti-SSB antibodies (Table 1).

**Table 1:** Patient's Clinical Laboratory Findings BUN: Blood Urea Nitrogen; C3: Complement Component 3; C4: Complement Component 4; Anti-dsDNA: Anti-double strand DNA Antibodies; Anti-Sm: Anti-Smith Antibody; Anti-RNP: Anti-Ribonucleoprotein Antibody; Anti-Ro52/SSA: Anti-Ro-52 Antibody; Anti-La/SSB: Anti-La-Antibody; IgG: Immunoglobulin G; IgA: Immunoglobulin A; IgM: Immunoglobulin M.

Variable	Admission	Reference Range
Hemoglobin (g/dL)	3.2	12.5 – 15.3
Hematocrit (%)	10	36 – 48
Mean corpuscular volume (fL)	121	80 - 94
Platelets (cells/mm^3^)	323,000	150,000 – 445,000
Leukocytes (cells/mm^3^)	8,540	4,000 – 12,000
Neutrophils (cells/mm^3^)	5,120	1,600 – 6,000
Lymphocytes (cells/mm^3^)	1,537	600 – 1,400
Monocytes (cells/mm^3^)	470	800 – 1000
Eosinophils (cells/mm^3^)	133	300 – 500
Reticulocytes (%)	24%	1 – 3
Erythrocyte sedimentation rate (mm/h)	105	0 – 25
Lactate dehydrogenase (U/L)	550	205 – 350
Total bilirubin (mg/dL)	2.4	0.5 – 1.5
Indirect bilirubin (mg/dL)	1.9	0.3 – 0.6
Haptoglobin (mg/dL)	< 10	10 – 250
Creatinine (mg/dL)	1.48	0.6 – 1.2
BUN (mg/dL)	32	8 – 25
24-hour-proteinuria (mg/24 hrs)	7500	120 – 180
C3 (mg/dL)	68	90 – 180
C4 (mg/dL)	4	10 – 40
Direct Coombs Test	+++	Negative
Monospecific Direct Coombs Test	C3d +++ IgG -	Negative
Cold agglutinins	Positive 1: 256	Negative
Anticellular antibodies Hep-2	Positive 1:3200 AC-4 / AC-5	Negative
Anti-dsDNA	14.6	Negative 0 - 20
Anti-Sm	4.2	Negative 0 - 20
Anti-RNP	5.25	Negative 0 - 20
Anti-Ro52/SSA	256.4	Negative 0 - 20
Anti-La/SSB	119.2	Negative 0 - 20
IgG (g/L)	1.5	0.8 – 1.6
IgA (g/L)	0.9	0.8 – 1.1
IgM (g/L)	2.42	0.5 – 1.4
Troponin I (pg/mL)	1163	0 – 14

**Figure 1:**
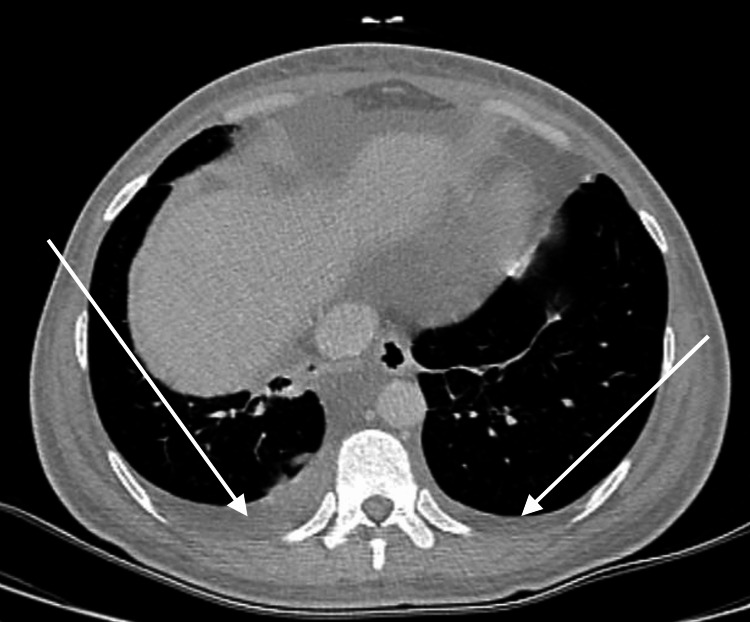
Patient's Thoracic CT Scan Revealing Bilateral Moderate Pleural Effusions (Arrows)

Left ventricle concentric hypertrophy and small pericardial effusion were noticed during the transthoracic echocardiogram, as well as bilateral pleural effusion without any other abnormalities in abdominal and chest CT scanning (Figure 1).

**Figure 2:**
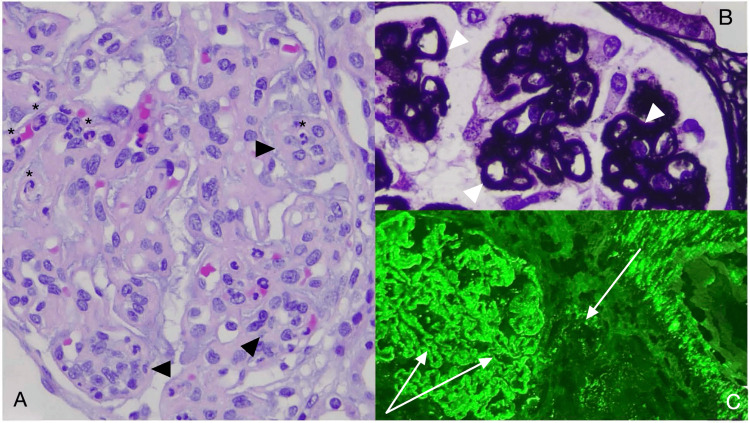
Renal Biopsy with Light Microscopic and Immunofluorescence Findings of ISN/RPS Classes IV and V Lupus Nephritis A: Global endocapillary hypercellularity (black arrowheads) with neutrophils (*) (Hematoxylin-Eosin, original magnification: 400x). B: Glomerular capillary wall thickening with argyrophilic subepithelial spikes (white arrowheads) (Jones Silver Stain, original magnification: 400x). C: Glomerular (i.e., mesangium and capillary wall) and extra-glomerular (i.e., arterial wall) fine granular deposits of IgG (white arrows) (FITC, original magnification: 200x).

Based on the clinical presentation and laboratory studies, SLE was suspected. A renal biopsy was performed, with renal histology confirming the diagnosis, identifying ISN/RPS Class IV and Class V LN (Figure 2), thus satisfying the ACR/EULAR 2019 SLE Classification Criteria. The patient was started on oral hydroxychloroquine with steroid tapering and intravenous cyclophosphamide following the Eurolupus protocol. Patient follow-up demonstrated complete remission of hemolysis with hemoglobin-level normalization and an adequate renal response with a rapid contraction in proteinuria at six months.

## Discussion

Cold agglutinins (CAs) are autoreactive IgM antibodies that agglutinate RBCs through antigen-antibody reactions that occur optimally at 0-4°C. At distal circulatory segments, CAs bind to the erythrocyte surface inducing complement fixation and activation through the classical pathway, as the red blood cell re-enters the warmer central circulation, CAs detach leaving behind C3b-opsonized RBCs that experience extravascular hemolysis through mononuclear phagocytosis in the liver [4]. The production of CAs is associated with a monoclonal lymphoproliferative disorder called cold agglutinin disease or it may be secondary to infection, lymphomas, and systemic autoimmune disorders composing the broader CAS [13].

CAS is exceedingly rare in SLE patients, being only sporadically reported in the literature. Medical database research through PubMed and Ovid revealed seven other cases (Table 2). All previously reported cases have described its occurrence in female patients of multiple ethnic backgrounds, with ages ranging from 17 to 74 years. Most cases share similar clinical and laboratory characteristics with our case, as a majority developed symptomatic anemia characterized by fatigue and exertional dyspnea, with accompanying symptoms such as fever and arthralgia; lymphadenopathy and/or splenomegaly are also common findings in these reported cases. Similarly, severe anemia is frequent, and positive DAT with elevated CAs is a universal finding in this condition. On the other hand, no other reported case has demonstrated concurrent nephritis or anti-Ro antibodies as patients predominantly show anti-dsDNA positivity [3,14-19]. Adequate steroid responses are a constant finding, with a minority of cases developing refractory disease which requires Rituximab treatment.

**Table 2:** Cold Agglutinin Syndrome Associated With SLE Case Reports ESR: Erythrocyte Sedimentation Rate; DAT: Direct Antiglobulin Test; ANA: Antinuclear Antibodies; Anti-dsDNA: Anti-double Stranded DNA Antibodies; SLE: Systemic Lupus Erythematosus; Anti-RNP: Anti-Ribonucleoprotein Antibodies; Anti-Sm: Anti-Smith Antibodies.

Case	Patient Characteristics	Clinical Manifestations	Laboratory Data	Treatment
Srinivasan et al. (2010) [3]	27-year-old African American female	Exertional dyspnea, fatigue, night sweats, generalized lymphadenopathy.	Hemoglobin: 8.3 g/dL, ESR > 100, positive DAT, elevated cold agglutinins, positive ANA, positive Anti-dsDNA, and hypocomplementemia	Remission after oral prednisone
Chaubey and Chhabra (2013) [14]	42-year-old Caucasian female	Exertional dyspnea, fatigue, arthralgia, photosensitivity, and splenomegaly.	Hemoglobin: 6.6 g/dL, positive DAT, elevated cold agglutinins, Negative ANA, Positive Anti-dsDNA and anti-cardiolipin antibodies, and hypocomplementemia	Poor response to intravenous steroids. Remission after Rituximab
Mohanty et al. (2019) [15]	17-year-old Indian female	Acute fever, arthralgias, generalized lymphadenopathy, hepatosplenomegaly, and a pulmonary consolidation.	Hemoglobin: 5.1 g/dL, positive DAT, elevated cold agglutinins, positive ANA, positive Anti-dsDNA, and hypocomplementemia	Broad-spectrum antibiotics. Remission after Intravenous methylprednisolone with oral steroid tapering
Kotani et al. (2006) [16]	53-year-old Japanese female	Known SLE history. Dyspnea, fatigue, and pallor.	Hemoglobin: 7.9 g/dL, positive DAT, elevated cold agglutinins, positive ANA, positive Anti-dsDNA, and hypocomplementemia	Refractory disease to intravenous methylprednisolone, cyclosporine and plasmapheresis. Remission induction after Rituximab treatment.
Nair et al. (1997) [17]	34-year-old Indian female	Pallor, polyarthritis, fever, alopecia, oral ulcers, and splenomegaly.	Hemoglobin: 6.0 g/dL, ESR > 100, positive DAT, elevated cold agglutinins, positive LE cells, and anti-dsDNA antibodies.	Remission after oral prednisolone with steroid tapering
Nagahata, Suzuki, and Takahashi (2023) [18]	74-year-old Japanese female	Known SLE history, increasing fatigue and Raynaud phenomenon	Hemoglobin: 5.3 g/dL, positive DAT, and elevated cold agglutinins	Blood transfusion. Cold avoidance. Rapid warming.
Osorio-Toro et al., 2023) [19]	22-year-old Colombian patient	Intermittent fever, fatigue, pallor, diarrhea, emesis, jaundice, lymphadenopathy, rash, peripheral edema, and synovitis.	Hemoglobin: 6.2 g/dL, ESR > 100, positive DAT, hypocomplementemia, positive ANA, Anti-RNP and Anti-Sm antibodies, elevated cold agglutinins. Kidney biopsy with Class II Lupus nephritis	High-dose dexamethasone, hydroxychloroquine, and mycophenolate mofetil.

Regarding treatment, available data suggests that corticosteroids tend to be suboptimal during the treatment of CAS as less than 20% of treated patients reach remission, and sustained response is associated with unacceptably high maintenance doses [20]. Similarly, there is scarce data concerning unspecific immunosuppressants; nonetheless, they seem to be generally ineffective. Directed monoclonal therapies such as Anti-CD20 (rituximab), Anti-C5 (eculizumab), and Anti-C1s (sutimlimab) have demonstrated promising results in primary CAS. Other measures such as cold avoidance, blood transfusion, and apheresis during critical situations are also accepted [16,17].

## Conclusions

The protean nature of SLE manifestations is ever so surprising. Secondary CAS associated with SLE is an extraordinarily rare disease feature that tends to be associated with acute-onset severe anemia, lymphadenopathy, and systemic manifestations such as fever, synovitis, nephritis, and elevated anti-dsDNA titers.

There is insufficient data to support a specific treatment algorithm; nonetheless, analogously to primary CAS management, anti-CD20 therapy appears to be one of the mainstays in disease control. 
